# Nanopore sequencing dataset of marine rocky shoreline microbial communities from the UK’s first marine citizen science week event

**DOI:** 10.1016/j.dib.2026.112966

**Published:** 2026-06-11

**Authors:** Daniele De Corte, Nathan Hubot, Christophe Patterson, Ben Holt, Stephanie Mills, Spencer Long, Jonathan S. McQuillan

**Affiliations:** a Ocean Technology and Engineering, The National Oceanography Centre, European Way, Southampton, SO14 3ZH, UK; b The Rock Pool Project, Marine Biological Association, Citadel Hill, Plymouth, PL1 2PB, UK; c Natural England, Foss House, Kings Pool, 1-2 Peasholme Green, York, YO1 7PX, UK; d Environmental Sequencing Facility, University of Southampton, Waterfront Campus, European Way, Southampton, SO14 3ZH, UK

**Keywords:** Metabarcoding, Nanopore sequencing, Citizen science, Marine, Biodiversity

## Abstract

In March of 2025 the UK’s first Marine Citizen Science Week recruited Citizen Scientists from around the country to participate in a series of activities exploring the ecology and biodiversity of rocky shorelines at sites on the northeast and southwest coastlines of England. To investigate the ‘unseen’ microbial biodiversity the organisers instructed participants to collect microorganisms from seawater using sterile pressure driven filtration units; these were sent to a laboratory for DNA extraction and metabarcoding analysis. DNA was extracted from material collected on the filter membranes and used in PCR amplification to generate 16S rRNA gene amplicons (bacterial) and 18S rRNA gene amplicons (protozoal). Each amplicon was ligated with a unique barcode and compiled into sequencing libraries, which were sequenced using Oxford Nanopore Technologies’ MinION platform. The data sets for 16S rRNA gene amplicon sequencing and 18S rRNA gene amplicon sequencing reads have been uploaded as raw fastq files to the publicly accessible NCBI Sequencing Read Archive. The data sets provide an overview of the bacterial and eukaryotic microbial communities present in seawater collected from two geographically distinct rocky shore environments.

Specifications Table

**Table:** 

Subject	Biology
Specific subject area	Microbial community and biodiversity, metabarcoding sequencing data from rocky shorelines on the northeast and southwest coasts of England.
Type of data	Raw FASTQ files.
Data collection	Microbial community samples were collected on filter membranes from seawater. DNA was extracted directly from the filters and amplified by PCR. 16S rRNA gene amplicons and 18S rRNA gene amplicons were barcoded for multiplex sequencing using Oxford Nanopore Technologies’ MinION sequencing system.
Data source location	Microbial samples were collected from rocky shorelines near to Plymouth Queen Anne’s Battery and within the Whitley Bay area, located on the southwest and northeast English coastlines respectively. Sample DNA extraction, PCR and sequencing were carried out by the National Oceanography Centre, Southampton, UK.
Data accessibility	Repository name: NCBI Sequence Read Archive (SRA) Data identification number: ID 1436507; ID 1436513 Direct URL to data: https://www.ncbi.nlm.nih.gov/bioproject/PRJNA1436507; https://www.ncbi.nlm.nih.gov/bioproject/PRJNA1436513
Related research article	None

## 1 Value of the Data

• The data is a contribution to marine microbial phylogenetic resources and the characterisation of the bacterial and eukaryotic microbial communities in rocky shore ecosystems with potential applications for the study and assessment of shoreline biodiversity and species composition.
• The data can be reused in studies of marine microbial ecology and could be employed as a baseline for the assessment of impacts to rocky shore ecosystem health such as pollutants or climate-related hazards such as extreme weather events.
• Although only two sampling locations were used to prepare the dataset, each site was sampled multiple times, and the data collected is a comprehensive assessment of microbial community diversity at each location.
• There is a paucity of existing data to describe the microbial biodiversity of rocky shorelines. The dataset presented here could be valuable for consolidation with, and comparison with, further studies of these highly heterogeneous and ecologically important environments.

## 2 Background

Intertidal marine rocky shorelines are exceptionally heterogeneous environments with daily extremes in physical and chemical parameters that impose the selection of remarkable adaptations and ecological traits. Therefore, the popular pastime of exploring rocky shorelines represents a valuable opportunity to educate and excite young and old people alike about biodiversity and ecology, as well as providing a means to survey and monitor the impact of climate change and pollution on these important ecosystems. The dataset presented here was generated during the first UK Marine Citizen Science Week, which took place in March 2025, supported by the Defra-funded marine Natural Capital and Ecosystem Assessment program. The objective was to increase public awareness and education of the importance of shoreline biodiversity and ecology through recruiting ‘Citizen Scientists’ to undertake ecological surveys, focusing on two rocky shoreline environments on the southwest and northeast coasts of England. This included the collection of microbial samples by filtering seawater, followed by a scientist-led DNA metabarcoding-based assessment of the bacterial and protozoal biodiversity using Nanopore sequencing. The dataset is raw and processed reads of 16S rRNA gene amplicon sequencing (bacterial) and 18S rRNA gene amplicon sequencing (protozoal/eukaryote) using DNA extracted from filter membranes. This data can be valuable for further studies of rocky shoreline ecosystem biodiversity and change.

## 3 Data Description

Metabarcoding sequencing of 16S rRNA gene amplicons and 18S rRNA gene amplicons generated from DNA extracted from the filtrate from seawater samples was carried out by the National Oceanography Centre (UK) using the Oxford Nanopore Technologies MinION platform. A total of 41 16S rRNA gene amplicons and 40 18S rRNA gene amplicons were sequenced to observe the microbial biodiversity. The 16S rRNA gene amplicons were prepared in 3 individual libraries (‘run 1′ (samples 1–16), ‘run 2′ (samples 17–32) and ‘run 3′ (samples 33–41), each library was sequenced on a ‘Flongle™’ Flow Cell. The 18S rRNA gene amplicons were prepared as a single library incorporating all samples and sequenced on a standard flow cell.

The raw sequence reads have been deposited into the publicly accessible Sequencing Read Archive (SRA) database at the National Centre for Biotechnology Information (NCBI) with the BioProject accessions PRJNA1436513 for 16S rRNA gene amplicon sequencing and PRJNA1436507 for 18S rRNA gene amplicon sequencing. Table 1, Table 2 display the file names and location of where the samples were collected for 16S rRNA gene amplicon sequencing and 18S rRNA gene amplicon sequencing respectively. Additional meta data is linked to the deposited data sets at NCBI.

**Table 1:** Sequencing file information for 16S rRNA gene amplicon sequencing (Bacterial) data.

*Location*	*Sequencing Run*	*Barcode^1^*	*Filename*	*SRA Accession*
Whitley Bay	1	1	barcode01_combined_r1.fastq.gz	SRS28372109
Whitley Bay	1	2	barcode02_combined_r1.fastq.gz	SRS28372110
Whitley Bay	1	3	barcode03_combined_r1.fastq.gz	SRS28372121
Whitley Bay	1	5	barcode05_combined_r1.fastq.gz	SRS28372131
Whitley Bay	1	6	barcode06_combined_r1.fastq.gz	SRS28372142
Whitley Bay	1	7	barcode07_combined_r1.fastq.gz	SRS28372147
Whitley Bay	1	8	barcode08_combined_r1.fastq.gz	SRS28372145
Whitley Bay	1	9	barcode09_combined_r1.fastq.gz	SRS28372146
Whitley Bay	1	10	barcode10_combined_r1.fastq.gz	SRS28372148
Whitley Bay	1	11	barcode11_combined_r1.fastq.gz	SRS28372149
Whitley Bay	1	12	barcode12_combined_r1.fastq.gz	SRS28372112
Whitley Bay	1	13	barcode13_combined_r1.fastq.gz	SRS28372111
Whitley Bay	1	14	barcode14_combined_r1.fastq.gz	SRS28372114
Whitley Bay	1	15	barcode15_combined_r1.fastq.gz	SRS28372113
Whitley Bay	1	16	barcode16_combined_r1.fastq.gz	SRS28372115
Whitley Bay	2	1	barcode01_combined_r2.fastq.gz	SRS28372117
Plymouth	2	2	barcode02_combined_r2.fastq.gz	SRS28372116
Whitley Bay	2	3	barcode03_combined_r2.fastq.gz	SRS28372118
Plymouth	2	4	barcode04_combined_r2.fastq.gz	SRS28372119
Whitley Bay	2	5	barcode05_combined_r2.fastq.gz	SRS28372120
Whitley Bay	2	6	barcode06_combined_r2.fastq.gz	SRS28372122
Plymouth	2	7	barcode07_combined_r2.fastq.gz	SRS28372123
Plymouth	2	8	barcode08_combined_r2.fastq.gz	SRS28372124
Plymouth	2	9	barcode09_combined_r2.fastq.gz	SRS28372125
Plymouth	2	10	barcode10_combined_r2.fastq.gz	SRS28372126
Plymouth	2	11	barcode11_combined_r2.fastq.gz	SRS28372128
Plymouth	2	12	barcode12_combined_r2.fastq.gz	SRS28372127
Plymouth	2	13	barcode13_combined_r2.fastq.gz	SRS28372129
Plymouth	2	14	barcode14_combined_r2.fastq.gz	SRS28372130
Plymouth	2	15	barcode15_combined_r2.fastq.gz	SRS28372132
Plymouth	2	16	barcode16_combined_r2.fastq.gz	SRS28372134
Plymouth	3	1	barcode01_combined_r3.fastq.gz	SRS28372133
Plymouth	3	2	barcode02_combined_r3.fastq.gz	SRS28372135
Whitley Bay	3	3	barcode03_combined_r3.fastq.gz	SRS28372136
Plymouth	3	4	barcode04_combined_r3.fastq.gz	SRS28372137
Plymouth	3	5	barcode05_combined_r3.fastq.gz	SRS28372138
Whitley Bay	3	6	barcode06_combined_r3.fastq.gz	SRS28372139
Plymouth	3	7	barcode07_combined_r3.fastq.gz	SRS28372140
Plymouth	3	8	barcode08_combined_r3.fastq.gz	SRS28372141
Plymouth	3	9	barcode09_combined_r3.fastq.gz	SRS28372143
Plymouth	3	10	barcode10_combined_r3.fastq.gz	SRS28372144

**Table 2:** Sequencing file information for 18S rRNA gene amplicon sequencing (Protozoal) data.

*Location*	*Sequencing Run*	*Barcode^1^*	*Filename*	*SRA Accession*
Plymouth	1	1	barcode01_combined.fastq.gz	SRS28371827
Plymouth	1	2	barcode02_combined.fastq.gz	SRS28371828
Plymouth	1	3	barcode03_combined.fastq.gz	SRS28371840
Plymouth	1	4	barcode04_combined.fastq.gz	SRS28371829
Plymouth	1	5	barcode05_combined.fastq.gz	SRS28371841
Plymouth	1	6	barcode06_combined.fastq.gz	SRS28371842
Plymouth	1	7	barcode07_combined.fastq.gz	SRS28371835
Plymouth	1	8	barcode08_combined.fastq.gz	SRS28371838
Plymouth	1	9	barcode09_combined.fastq.gz	SRS28371843
Plymouth	1	10	barcode10_combined.fastq.gz	SRS28371830
Plymouth	1	11	barcode11_combined.fastq.gz	SRS28371831
Plymouth	1	12	barcode12_combined.fastq.gz	SRS28371824
Plymouth	1	13	barcode13_combined.fastq.gz	SRS28371839
Plymouth	1	14	barcode14_combined.fastq.gz	SRS28371826
Plymouth	1	15	barcode15_combined.fastq.gz	SRS28371832
Plymouth	1	16	barcode16_combined.fastq.gz	SRS28371844
Plymouth	1	17	barcode17_combined.fastq.gz	SRS28371813
Plymouth	1	18	barcode18_combined.fastq.gz	SRS28371845
Plymouth	1	19	barcode19_combined.fastq.gz	SRS28371814
Plymouth	1	20	barcode20_combined.fastq.gz	SRS28371837
Plymouth	1	21	barcode21_combined.fastq.gz	SRS28371833
Plymouth	1	22	barcode22_combined.fastq.gz	SRS28371825
Plymouth	1	23	barcode23_combined.fastq.gz	SRS28371836
Plymouth	1	24	barcode24_combined.fastq.gz	SRS28371846
Whitley Bay	1	25	barcode25_combined.fastq.gz	SRS28371847
Whitley Bay	1	26	barcode26_combined.fastq.gz	SRS28371848
Whitley Bay	1	27	barcode27_combined.fastq.gz	SRS28371849
Whitley Bay	1	28	barcode28_combined.fastq.gz	SRS28371850
Whitley Bay	1	29	barcode29_combined.fastq.gz	SRS28371851
Whitley Bay	1	30	barcode30_combined.fastq.gz	SRS28371815
Whitley Bay	1	31	barcode31_combined.fastq.gz	SRS28371816
Whitley Bay	1	32	barcode32_combined.fastq.gz	SRS28371817
Whitley Bay	1	33	barcode33_combined.fastq.gz	SRS28371818
Whitley Bay	1	34	barcode34_combined.fastq.gz	SRS28371819
Whitley Bay	1	36	barcode36_combined.fastq.gz	SRS28371820
Whitley Bay	1	37	barcode37_combined.fastq.gz	SRS28371834
Whitley Bay	1	38	barcode38_combined.fastq.gz	SRS28371821
Whitley Bay	1	39	barcode39_combined.fastq.gz	SRS28371822
Whitley Bay	1	40	barcode40_combined.fastq.gz	SRS28371823

Table 3 presents the statistics of the sequencing datasets obtained from 16S rRNA gene amplicon sequencing and 18S rRNA gene amplicon sequencing of rocky shore microbial communities, including the number of samples (N_Samples), operational taxonomic units (N_OTUs), total sequences (Total_seqs), and sequencing depth per sample (Min_seqs, Max_seqs, Mean_seqs, Median_seqs).

**Table 3:** Sequencing Statistics.

	N_Samples	N_OTUs	Total_seqs	Min_seqs	Max_seqs	Mean_seqs	Median_seqs
**16S rRNA gene amplicon Sequencing**	41	539	515402	4351	250772	12570	11175
**18S rRNA gene amplicon sequencing**	40	179	273340	49	20075	7008	5725

**Fig. 1:**
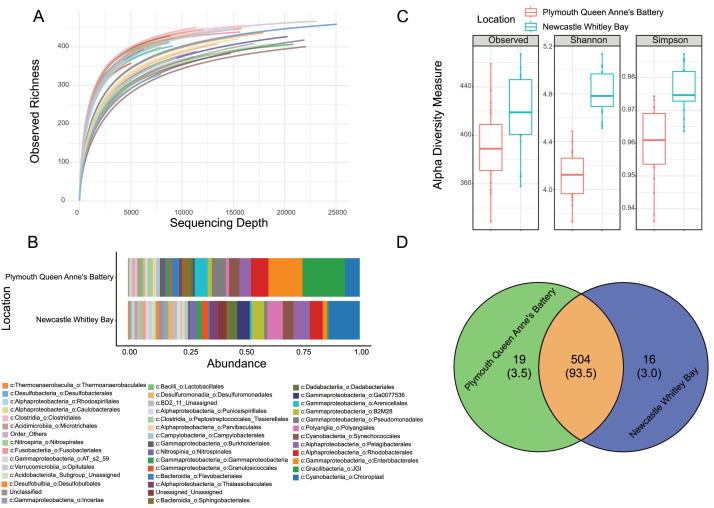
Rarefaction curves based on 16S rRNA gene amplicon sequences of the rockpool bacterial communities obtained from the different samples (A). Relative contribution of the most abundant bacterial orders to the total number of 16S rRNA gene amplicon sequences from the two locations (B). Box plots showing richness, Shannon diversity index, and Simpson evenness index for the bacterial communities from the two sampled locations (C). Venn diagram showing the shared and unique bacterial operational taxonomic units (OTUs) between the two locations (D).

**Fig. 2:**
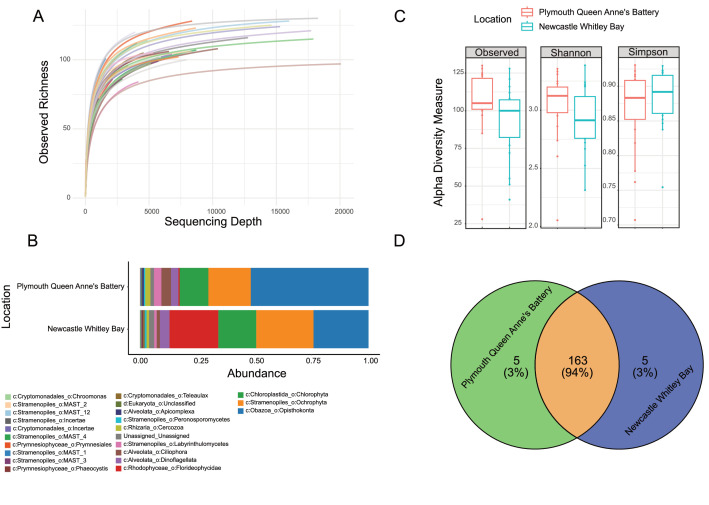
Rarefaction curves based on 18S rRNA gene amplicon sequences of the rockpool eukaryotic communities obtained from the different samples (A). Relative contribution of the most abundant eukaryotic orders to the total number of 18S rRNA gene amplicon sequences from the two locations (B). Box plots showing richness, Shannon diversity index, and Simpson evenness index for the eukaryotic communities from the two sampled locations (C). Venn diagram showing the shared and unique eukaryotic operational taxonomic units (OTUs) between the two locations (D).

Fig. 1, Fig. 2 help to describe the sequencing datasets generated for the 16S rRNA gene amplicons and 18S rRNA gene amplicons, respectively.

## 4 Experimental Design, Materials and Methods

### 4.1 Sample collection

Samples were collected during low tide on the 3rd of March 2025, by citizen scientists working along two rocky-shoreline locations near to Plymouth Queen Anne’s Battery (50° 21′ 56.8152″N 4° 7′ 52.2258″W) and within Whitley Bay (55°02′44.3″N 1°26′25.6"W), respectively on the northeast and southwest English coastlines. The participants were instructed to collect a sample by filling a 50mL-capacity plastic syringe with seawater and pushing the water through a Sterivex™ (Merck, UK) filter unit via a Luer-Lock type connection. The filter units had a 0.22-micron pore size polyether sulfone membrane and were provided in Sterile packaging. During the activity, Citizen Scientists were instructed to repeat the sampling process until encountering strong resistance (the filter was blocked) or until 500 mL (10x passes) had been processed. The participants were instructed to remove as much liquid from the filter ‘hold-up’ volume as possible by ‘chasing’ the liquid out with air under gravity. Filter units were sealed inside sterile plastic tubes and shipped with ice packs to the National Oceanography Centre in Southampton (UK) for analysis. 62 samples were provided.

### 4.2 DNA extraction

DNA was extracted and purified using the DNeasy PowerWater Sterivex Kit (Qiagen, UK) according to the manufacturer’s recommended protocol. The recovered DNA was eluted in 50 μL of sterile, nuclease-free water and stored at −70 °C until required. DNA yield and purity were assessed using a Nanodrop spectrophotometer and a Qubit fluorimeter with the Qubit HS Assay Kit (ThermoFisher Scientific, UK), using the manufacturer’s recommended protocol. Of 62 samples provided, 42 were successfully extracted to yield DNA of suitable quality for analysis.

### 4.3 Polymerase chain reaction

DNA metabarcoding analysis was done by 16S rRNA gene amplicon sequencing (for bacteria) and 18S rRNA gene amplicon sequencing (for protozoal) genes, which were first amplified by PCR. General (‘universal’) primers were used to generate a 1262 bp 16S rRNA gene amplicon, and a 1438 bp 18S rRNA gene amplicon; primer sequences are shown in Table 4.

**Table 4:** Primer sequences.

Primer Name	Target Gene	Sequence	References
16S 27F	16S	AGAGTTTGATCMTGGCTCAG	[9] Weisberg et al. (1991)
16S 1289R	16S	ACTAAGAACGGCCATGCACC	[10] Hadziavdic et al. (2014)
18S 82F	18S	GAAACTGCGAATGGCTC	[11] Lopez-Garcia et al. (2003)
18S 1520R	18S	CYGCAGGTTCACCTAC	

PCR reactions were prepared using the GoTaq G2 DNA Polymerase system (Promega, UK) with modifications to the manufacturer’s suggested protocol to improve yield. Each reaction contained GoTaq® Colourless PCR Buffer at the manufacturer’s recommended concentration, which included 1 mmol.L^−1^ of MgCl_2_, 0.5 mmol.L^−1^ each of dATP, dTTP, dCTP and dGTP, 400 nmol L^−1^ of each primer, 50 μg of BSA (Life Technologies, UK), 1 U GoTaq G2 DNA polymerase and 0.5 μL of extracted DNA sample; the total volume was made to 50 μL using nuclease-free water. The reactions were prepared in 0.2 mL nuclease‐free polycarbonate tubes (Applied Biosystems, UK) and incubated using a Thermal cycler (Mycycler, Bio-rad UK). For 16S rRNA gene amplification, the thermal cycling conditions were 95 °C for 2 min followed by 35 cycles of 95 °C for 15 s, 58 °C for 10 s and 72 °C for 90 s, followed by a final extension at 72 °C for 10 min. For 18S rRNA gene amplification, the thermal cycling conditions were 95 °C for 2 min followed by 35 cycles of 95 °C for 15 s, 52 °C for 20 s and 72 °C for 90 s, followed by a final extension at 72 °C for 10 min.

Amplified DNA was analysed by agarose-TAE gel electrophoresis to confirm the presence of a single product of the correct size, followed by purification using the QiaQuick PCR Purification Kit (Qiagen, UK) according to the manufacturer’s recommended protocol and elution in 30 μL nuclease-free water. Purified amplicons were quantified using a Qubit and Qubit HS Assay Kit and stored at −20 °C.

### 4.4 Amplicon barcoding and preparation for nanopore sequencing

Purified PCR products were individually barcoded and ligated with sequencing adaptors for multiplexed sequencing using the Ligation Sequencing DNA Native Barcoding Kit 96 V14 (Oxford Nanopore Technologies, UK). Individual libraries were prepared for the 16S rRNA gene amplicons and the 18S rRNA gene amplicons. Samples were processed in batches to minimise loitering times during the workflow; the 16S rRNA gene amplicons were processed in 3 batches and sequenced individually; 18S rRNA gene amplicons were processed in 2 batches and then combined prior to sequencing. In general, the manufacturer’s recommended protocol was followed with some exceptions to improve the final library concentration and purity, which are described below. DNA repair and ‘end-prep’ was carried out using the NEBNext® UltraTM II End Repair/dA-Tailing Module (New England Biolabs, UK); each reaction contained at least 400 ng of PCR product (less where amplicon DNA concentration was too low), 2 μL UltraTM II End-prep reaction buffer and 1 μL UltraTM II End-prep enzyme mix; the final volume was 16 μL. Barcode ligation was carried out using the NEB Blunt/TA Ligase Master Mix (New England Biolabs, UK); each reaction contained 8 μL of end-prepared DNA, 2.5 μL of barcode mixture and 10 μL of Blunt/TA Ligase Master Mix; the final volume was 20.5 μL. Sequencing adapter ligation was carried out using the NEBNext® Quick Ligation Module (New England Biolabs, UK) wherein all steps requiring AMPure XP Beads were carried out using double the manufacturer’s recommended volumes. After pooling, all steps were carried out using 1.5 mL Eppendorf LoBind tubes (ThermoFisher Scientific, UK); the final library was eluted in 15 μL of the provided Elution Buffer and kept at 4 °C, sequencing was carried out immediately. The final library concentrations were estimated using a Qubit and Qubit HS Assay Kit.

### 4.5 Nanopore sequencing

Two types of Nanopore sequencing flow cell were used. 16S rRNA gene amplicons were prepared in 3 batches, and each library was sequenced individually using a Flongle™ (R10.4.1) flow cell on a MinION MK1B personal sequencing device (Oxford Nanopore Technologies, UK) according to the manufacturer’s recommended protocol. 18S rRNA gene amplicons were sequenced using a single MinION flow cell (R10.4.1) according to the manufacturer’s recommended protocol. Sequence data was recorded using MinKNOW version 25.05.12.

### 4.6 Sequence analysis

Reads containing the target primer sequences were identified using Cutadapt [1]. Reads with the correct primer pairs were subsequently quality filtered using NanoFilt [2]. Only sequences with the expected amplicon length and a quality score equal or greater than 15 were retained for downstream analyses. Additionally, reads were oriented and chimera were removed using VSEARCH [3]. High quality reads were clustered into groups of closely related sequences using AmpliconSorter [4]. Representative sequences from each cluster were then used to generate consensus sequences. Taxonomic assignments of the resulting consensus sequences were performed using VSEARCH [3] against the SILVA SSU rRNA reference database (release 138.2, 99%), including both 16S rRNA gene and 18S rRNA gene sequences. The operational taxonomic unit (OTU) table was generated using VSEARCH [3] by mapping quality filtered reads to the consensus sequences with a minimum identity threshold of 0.90. Detailed information on the bioinformatic pipeline is reported in GitHub (https://github.com/DanieleDeco/meta-barcoding-sequencing-from-rocky-shoreline.git). All statistical analyses and visualizations were performed in R (version 4.5.1) using the packages phyloseq [5], vegan [6], ggplot2 [7], and MicEco [8].

## Limitations

The dataset includes Nanopore sequencing data from microbial samples collected on filters by Citizen Scientist participants in a public engagement event. Whilst the participants were briefed and supervised by a professional ecologist during the sampling window, we cannot be certain of the robustness of the sample collection practices. The sampling technique and the precise amount of water filtered may have been variable between the participants. However, many replicate samples were collected from each site, and the overall microbial community diversity at each site was observed to be consistent across the replicate samples. Moreover, although the participants were instructed to wear sterile gloves and flush collection syringes prior to sampling, we cannot fully exclude the possibility of contaminant DNA sequences introduced at the point of sample collection.

## Ethics Statement

All the authors have read and follow the ethical requirements for publication in Data in Brief and confirm that the current work does not involve human subjects, animal experiments, or any data collected from social media platforms.

## CRediT Author Statement

**Daniele de Corte:** Methodology, Validation, Formal Analysis, Investigation, Data Curation, Writing – Review and Editing, Visualisation. **Nathan Hubot:** Methodology, Validation, Formal Analysis, Investigation, Resources, Data Curation, Writing – Review and Editing, Visualisation. **Christophe Patterson:** Conceptualisation, Methodology, Investigation, Writing – Review and Editing, Supervision, Project Administration. **Ben Holt:** Conceptualisation, Methodology, Investigation, Writing – Review and Editing, Supervision, Project Administration, Funding Acquisition. **Stephanie Mills:** Conceptualisation, Methodology, Investigation, Writing – Review and Editing, Supervision, Project Administration, Funding Acquisition. **Spencer Long:** Resources, Supervision. **Jonathan McQuillan:** Methodology, Validation, Formal Analysis, Investigation, Data Curation, Writing – Original Draft, Writing – Review and Editing, Supervision, Project Administration, Funding Acquisition.

## Data Availability

NCBI18S sequencing rock pool eukaryotes (Original data)
NCBI16S sequencing rock pool microbiomes (Original data)

## References

[1] Martin M. (2011). Cutadapt removes adapter sequences from high-throughput sequencing reads. EMBnet.journal.

[2] De Coster W., D’Hert S., Schultz D.T., Cruts M., Van Broeckhoven C. (2018). NanoPack: visualizing and processing long-read sequencing data. Bioinformatics.

[3] Rognes T., Flouri T., Nichols B., Quince C., Mahé F. (2016). VSEARCH: a versatile open source tool for metagenomics. PeerJ.

[4] Vierstraete A.R., Braeckman B.P. (2022). Amplicon_sorter: a tool for reference-free amplicon sorting based on sequence similarity and for building consensus sequences. Ecol. Evol..

[5] McMurdie P.J., Holmes S. (2013). Phyloseq: an R package for reproducible interactive analysis and graphics of microbiome census data. PLOS ONE.

[6] Oksanen J., Simpson G., Blanchet F., Kindt R., Legendre P., Minchin P., O'Hara R., Solymos P., Stevens M., Szoecs E., Wagner H., Barbour M., Bedward M., Bolker B., Borcard D., Borman T., Carvalho G., Chirico M., De Caceres M., Durand S., Evangelista H., FitzJohn R., Friendly M., Furneaux B., Hannigan G., Hill M., Lahti L., Martino C., McGlinn D., Ouellette M., Ribeiro Cunha E., Smith T., Stier A., Ter Braak C., Weedon J. (2026). Vegan: community Ecology package. https://vegandevs.github.io/vegan/.

[7] Wickham H. (2016).

[8] J. Russel, J. Oksanen (2025). Russel88/MicEco: v0.10.0 (v.0.10.0). Zenodo. 10.5281/zenodo.17241236.

[9] Weisburg W.G., Barns S.M., Pelletier D.A., Lane D.J. (1991). 16S ribosomal DNA amplification for phylogenetic study. J. Bacteriol..

[10] Hadziavdic K., Lekang K., Lanzen A., Jonassen I., Thompson E.M., Troedsson C. (2014). Characterization of the 18S rRNA gene for designing universal eukaryote specific primers. PLoS ONE.

[11] López-García P., Philippe H., Gail F., Moreira D. (2003). Autochthonous eukaryotic diversity in hydrothermal sediment and experimental microcolonizers at the Mid-Atlantic Ridge. Proc. Natl. Acad. Sci. U.S.A..

